# Function

**Published:** 1867-05

**Authors:** W. H. Atkinson


DENTAL REGISTER.

VOL. XXI.] MAY, 1867. [No. 5.

### Original Communications.

### FUNCTION.

BY W. H. ATKINSON, M. D., D. D. S.

FUNCTION is the *work* of body, therefore the body to which it belongs, must first be defined before we can make intelligible the particularity of the work or Function we propose to explain:

The body is the machine through which are elaborated those movements denominated Function by the power that sets up and continues the motion or motions which constitute Function.

So crude are all our apprehensions of biological processes, that close definition, under present classifications of ideas, language and beings, is next to an impossibility. And yet we see clearly enough, that every body capable of Function must have being; which opens the question of How bodies become or are constituted individualities?

As our ideas dominate all effort at thought, in whatever direction of our mental labor, it behooves us to look well to these, as we admit them to inweave themselves into our mentality, or we shall be without means of a clear admeasurement, by which to compare the unknown with the known, and accurately define the stages of apprehension of truism on the one hand, and dogmatism on the other, which are the extremes of mental propositions. The first announcement of any proposition must assume the aspect of dogmatism, where it remains until proved a verity; then it at once takes its only other normal status, among proved aphorism, which thereby becomes to all who are familiar therewith, simply a truism. To the novice, therefore, all is dogmatic in first actual pronouncement, no less than to open vision (perception) "matter of course."—(truism.)

Function, as usually defined, is an act so complex as to defy distinct definition, short of much preparation of classification, and arrangement of limitations, as to what constitutes its character.

Were we to pursue the subject in exhaustive analysis, so far as now possible to us, we would be compelled to consider it as a whole, as well as parts, whose joint agreements or disagreements constitute the normal and abnormal display of the complex activities involved in that which we denominate general Function.

This analysis leads us, not to the absolute, but to the attained limit of our understanding of what constitutes general and special Function.

The body as a whole may be regarded as a Sea, exactly analagous to the ocean, which is made up of its Northern and Southern, Eastern and Western members, with seas, bays, necks, estuaries, shoals and margins, without which in due relations to the great ocean bed, air, and other surroundings, there could be no *tides* constituting the general Function of ocean.

Tide is perceptible to all observers, in all bodies of salt water, but some dispute would arise were we to assert that it was present in *all* bodies of waters, with the variation of degree only.

In like manner do the superficials, deny function to all bodies less than or more ambiguous than the recognized divisions of organs constituting systems of organs, such as bones, muscles, vessels, glands nerves, &c., &c.; failing to perceive that each of these has also its primates by whose presence alone it elaborates the Function proper to each.

Thus, the question is narrowed down to the seen and the *unseen* limits of movements, rather than, as it should be, extended beyond these mere effects of that which is Function proper; which resolves itself really into the implicit obedience of a less to a greater force; quantity and quality being amplifications and tempic and spacic transpositions and aspects of the one great principle of life which produces and presides over all possibility of Function, mechanical, chemical and vital!

These three aspects of Motion or Function are involved in every movement, occult or open, simple or complicate, throughout the domain of organized being.

Hence when we say a certain movement is chemical, electric or other modification of currental expression of life, it is known that our meaning is that the motion is principally displayed in the aspect most apparent to the observer: one referring the action to the department with which he is most familiar, while another refers it to his accustomed interpretation.

We may have tolerably correct apprehension of our solar system, as a whole, and yet be very little acquainted with that of the planets constituting it.

And were there not a unitary law of Function, which existed in and governed all bodies, great or small, it might be well regarded as irrelevant to speak of the Function of Suns, and systems of planets in this place with hope of a patient hearing. But since spectral analysis has brought us into tangible contact with (and proved the presence of the predominant metals that constitute,) the far off stars, to the crudest apprehension, we are encouraged to give free rein to our ardent desire to know the *certainty*, that "all things differ but in degree," and that "each Function," (when understood)

"becomes the key to every other Function of every possible body." After our most extended and most minute survey of bodies in action, what has presented itself as the type of all Functional activity, as displayed in every body without a single exception? Answer.—That which is usually called "*breathing*" is this example of "unitary Function."

This is plain to all who are willing to take the pains to observe the essential movements of cells, tissues, organs and systems; mineral, vegetable or animal, throughout the entire range of these bodies, complex and simple.

If then inspiration (diastole), placement (digestion), and expiration (systole), be the generic acts constituting all of Function that is apparent to external observation, is it not conclusive that supply and waste, health and disease, are dependent upon the degree and manner of the performance of this triune role, the basis of Function everywhere!

That which is necessary to maintain a body in fit condition to perform its Functions, must be received from without in combination with much that is not only not needed, but incompatible to the well being of the body to be sustained by the nutrient elements sought in food. This involves separation, and election to appropriation or rejection of that which helps on the one hand, or hurts on the other.

It is this mysterious election and repulsion, which constitutes the basis of organic movement or Function, which, when we shall have comprehended, we shall be able to accommodate ourselves to this law of being, and thus inherit a perfectly blissful state of existence, that shall continue so long as the supply of the constituents of our being are within reach of appropriation!

This happy consummation is foreshadowed in the universal dread of death, and desire to live perpetually in some cognizable state of conscious existence, by all forms of individual being within our power of perception. If the Function of desire springs from *sense of need* spontaneously reaching out for something to satisfy, may we not as positively gather from the exercise of this universal passion to live, the proof that it will be gratified somewhere and sometime; as certainly as that the perfection of the organism, from which springs hunger, thirst and sexual passion, is the specific end for which this corelation of organs was called into being!

The first two of these have especial reference to the individual, so that it may become supplied with the necessary constituents of its own body expressly to enable it to store, after full self-satisfaction, an excess of the elements of being, for the purposes of multiplication, when the favoring conditions may contribute to this end of individual existence.

All that is necessary to prove the foregoing assertions, as to the end of life, is to take a survey of two groups of any species of domestic animals, the one nearly starved, and the other fed to repletion; or to compare the wild animals that live in a climate of unvarying temperatures, in which a constantly full supply of food is at hand, breeding at all seasons, without reference to the time of year, with the same species of animals, whose habit of indulgence in the reproductive instinct is governed by seasons of abundance of food, and the multiplicity or scarcity of the same varieties and species with whom to associate.

The ill fed have no desire, because they have a minus instead of a plus quantity of sun and earth presence stored within their proper bodies; while the well fed are in a furor of excitement, the moment they come within the sphere of influence of those having the opposite kind of vital endowment or presence, expressly that each may give to the other, that of which it has to spare, and thus equilibrize its own personality, by fulfillment of the highest planetary act, in introducing a new planet of the exact equation of endowments of its parent stocks.

All bodies in a state of diastole, induce currental movements toward their centers, from the most unpronounced and finest, to the most differenced and the coarsest examples of individual existences.

Thus we must accept the reciprocity existing among all bodies, whether it be pleasant to us or not, in the predominant stage of our development in which we are so prone to say *the sentiency* resides.

The fact is, the "me" resides in the whole organism, as is proved by the cognition of pain and pleasure in the remotest parts, even more keenly than in the brain itself, the boasted "especial seat" of *consciousness*:—

All well endowed beings must have association, whether that be what we call congruous or incongruous, depends upon the proximity of like or unlike bodies, with whom to intermarry, as instanced throughout civilized and savage life, among men no less than by the oft observed examples of the so called unnatural attachments among domestic beasts and birds.

Even Crusoe must talk to and associate with his goats, the rocks, the surf, and the inconstant wind in the absence of the *genus homo*. Association, consists in spiritual, mental and bodily nearness. Each of these three states or spheres has its specific predominant method and character of acting and being acted upon. We have been so long accustomed to say that associations were good or bad in character, that we have practically ignored the ONE government whose sway *can* be only good, by confining our observations to relational conditions, which induced us to say that association was good for one and bad for the other, in accordance with the view we took of the importance of the bodies in association.

If we have injurious spiritual association, "exorcism" is the cure; if mental darkness or deflections afflict us, "education" is the cure; and if incompatible or poisonous contacts (association) occur in our food, "purgation" is the means to restore the lost balance, so necessary to the enjoyment of our birthright, to be in the blissfull exercise of every function of our complex existence.

The origin of bodies is traceable to unseen currents, within an amorphous plastic mass.

These currental lines have been denominated the movements of light, heat and electricity. All these must be in predominant proportion above their manifestations elsewhere, to enable us to perceive them.

It has been said that we cannot cognize either of these agents, and that we measure their presence and operation by their effects on perceptible bodies. This is no anomaly in nature; for in the order of things, we know and measure the character and condition of all things alone, by a process called comparison. Thus, all our advances are but successful provings by comparison of the unknown to the known propositions, that have found lodgement within our mental receptacles.

The unitary source of these primal currents of force or power, has been proved to be the Sun, from whence they emanate, and toward which they tend in ultimate expression of movement, in completing not only this circuit, but all the Functions of separation and combination of all bodies in diastole and systole, as parts and as a whole. If then that which we denominate the suns ray be the embodiment of creative energy in direct and recurrent or deflected course for earth and every organism thereon: is not the Function of light sufficient to attract and retain our attention long enough to see that light is life to every creature capable of storing it for use in its proper body?

The ocean has been instanced as the great *Uterus*, which is in a state of *diastole* or inspiration of light, over that half of the globe which is constantly bathed in the sun presence, and *systole* or expiration on the opposite or dark side, thus establishing the *breathing*, or Function proper of the planet.

All terrestrial Function then, must be a due admixture of sun and earth processes, which are divisible into all possibility of phase of Function, from simple passivity of chaotic mass, to every variation of motion, segregate or aggregate, be it in mineral, vegetable or animal, in simplest or most complex correlation and culture.

Concurrent currental dominion obtains throughout every possibility of natural body, just as concurrent jurisdiction of municipal law holds the rein of government in communities of civilized men. They work together to the same end, viz.: the well-being of the individuals over whom they are exerted, no less than the perpetuity of the larger bodies they compose.

Hence, all the organs of the system under this dominion of supply, are bound by a tether of sympathy commensurate with the importance of each, to the whole and to each other, rendering health only predicable upon the exact balance of Function in each and in all.

This currental dominion in living bodies has its analogue in municipal communities in a force as intangible, and yet so potent as to be, like it, irresistable, which force is known by the name of "public opinion." This owes its power to concurrent consent of the mass of mind in the community to act together, without question of the propriety of their so acting. So soon as they hesitate, the charm is broken, and the public furor ends.

The Function of digestion, as a whole, as it occurs in terrestrial bodies, may be said to be the killing, complete and proper solving, and admixture in chaotic mass of the various foods upon which the particular body in which it occurs subsists. This is the prehension and due preparation of food by the body appropriating it.

But the act of appropriation (nutrition), depends upon the freedom of the body within its own especial atmosphere, from which it imbibes not only the food it seizes therein, but also the attenuated "sun presence," without which no act can take place. There are two aspects in which this is true, viz.: in *entire beings*, and in the constituent *cells* of which tissues and organs are composed; but it is true of organs only in a subordinate and modified sense.

In the entire being the cuticular and lung surfaces, are the points of *immediate* entrance of "sun presence," while cells are *mediately* supplied with the same, from the "mucoid sea," in which they are constantly bathed.

The absorption into cells of "infusorial mass," "granular mass," "living matter," "blood," "plasm," or "nutrient pabulum,"—these being the various names by which the prepared food is known—takes place at the point where it becomes completed, and ready for systemic use; whether that be in the alimentary tracts, vascular tracts or neural tracts, or in the extravascular sea of the juices out of which the cells are supplied with nutrient pabulum. Thus connective-cells become links in the chain of transmission of life-presence (sun presence) from the juices (in which it is stored), to the muscular fibrillæ for the purposes of muscular movements. In proof of which let me state that connective or areolar tissue constitutes the lines of limit to all primal bodies, whether they be cells of any variety or ultimate muscle fibrillæ, or disk, in which is elaborated and expressed the primal act of muscular Function.

If, then, digestion be a Function so subtle, it is evident, to be complete, each step or stage, in every locality must be fully and freely performed, to produce a blood stream, pure in character to sustain the body in health.

These steps or stages of preparation of blood from food are difficult of delineation, but may be said to occur in normal activity, distinctly pronounced, in the *mouth, stomach, duodeum, and intestines*; less distinctly seen in *veins*, in *absorbents* and in the *juices* of the flesh, or great "*sea of mucous mass*," and finally in the individual *cells*.

As before asserted, each body must be free to enable it to perform its specific Function. Now, that which constitutes this special freedom of bodies, is also the bond by which each is limited within a specific sphere of being and action, so as to afford an atmosphere not only to whole bodies, but also to the primates (cells), which constitutes them. Now, this inter-dependent bond of *service* and *freedom*, as a whole, is the complete expression of healthy or normal Function, while any excess of individual freedom, or any demand of excess of service from individuals, inaugurates unhealthy, or abnormal exercise of Function, which is the inception of disease.

These, then, are the principles with which we must become acquainted, before we can have adequate conception of what constitutes "Pathology," or that which belongs to Surgery, which is one of the many means to which medical men resort, to remove pathological manifestations from the bodies in which we see them displayed.

The details of an intelligent "Surgery," are as various as the functional deflections that necessitate their adoption. Hence, he who has the conception of that which constitutes normal Function, will be capable to detect abnormal activity, no less than foresee the course of procedure requisite to remove the latter, and re-establish the former. The more complete our knowledge of principles, the more beneficient and certain will our practices be.

